# EGR1 interacts with DNMT3L to inhibit the transcription of miR‐195 and plays an anti‐apoptotic role in the development of gastric cancer

**DOI:** 10.1111/jcmm.14597

**Published:** 2019-09-12

**Authors:** Yang Yang, Fei Wu, Jing Zhang, Ruifang Sun, Fang Li, Yulong Li, Su’e Chang, Lumin Wang, Xiaofei Wang, Liying Liu, Chen Huang

**Affiliations:** ^1^ School of Public Health Xi'anJiaotong University Health Science Center Xi'an China; ^2^ Department of Cell Biology and Genetics, School of Basic Medical Sciences Xi'anJiaotong University Health Science Center, Xi'an Jiaotong University Xi'an China; ^3^ Department of Clinical Medicine Medical College of Yan'an University Yan'an Shaanxi China; ^4^ Department of Pathology, School of Basic Medical Sciences Xi'anJiaotong University Health Science Center, Xi'an Jiaotong University Xi'an China; ^5^ Department of gastroenterology Shaanxi provincial people's hospital Xi’an China; ^6^ Department of Orthopedics Second Affiliated Hospital of Xi'an Jiaotong University Xi’an China

**Keywords:** AKT3, apoptosis, DNMT3L, EGR1, miR‐195

## Abstract

EGR1 regulates the expression of its downstream target genes and may exert different biological effects in different tumours. We found that the expression of EGR1 was increased in gastric cancer (GC), and silencing the expression of EGR1 promoted the apoptosis of GC cells. Moreover, overexpression of EGR1 repressed the apoptosis of GC cells. Bioinformatics analysis showed that EGR1 had binding sites at the upstream promoter region of miR‐195; ChIP assays were applied to determine EGR1 occupancy of the miR‐195 promoter. The RT‐PCR results showed that EGR1 suppressed the expression of miR‐195. The mechanism by which EGR1 acts as a transcriptional repressor is still unclear. Bioinformatics analysis showed that EGR1 may interact with DNMT3L. We confirmed that EGR1 and DNMT3L formed a complex, and EGR1 was an important player in the transcriptional control of miR‐195. Overexpression of miR‐195 inhibited proliferation and promoted apoptosis in GC cells. We found a well‐matched miR‐195 binding site at the AKT3 3′‐UTR. Double luciferase reporter assays showed that AKT3 was a target of miR‐195, and silencing AKT3 repressed cell proliferation and promoted apoptosis. Our results indicated EGR1 may interact with DNMT3L to inhibit the miR‐195‐AKT3 axis and regulate the GC cell apoptosis.

## 1 INTRODUCTION

Gastric cancer (GC) is one of the most common tumours worldwide. The number of newly diagnosed gastric cancer cases in China is about 679 100 per year, accounting for 15.8% of all new cancer cases. Gastric cancer ranks second in the morbidity and mortality of cancers,^1^ thus representing one of the malignant tumours that has the capacity to seriously threaten human health. Investigation of the molecular mechanism in gastric cancer development has great significance in reducing the incidence of gastric cancer and improving the prognosis of patients with gastric cancer.

The development of gastric cancer involves many factors such as the activation of oncogenes, inactivation of tumour suppressor genes, epigenetic regulation and abnormal expression of transcription factors. Transcription factors play key roles in cancer cell proliferation, apoptosis, invasion and metastasis by regulating downstream target genes. A variety of transcription factors are involved in the development of gastric cancer, including RUNX3,^2^ GrhL2,^3^ E2F1^4^ and EGR1.^5^

EGR1 as a member of early growth response (EGR) family that regulates the expression of its downstream target genes and plays a key role in cell growth, proliferation, apoptosis, migration, invasion and other physiological processes. EGR1 may exert different biological effects in different tumours. For example, in breast cancer,^6^ bladder cancer^7^ and lung cancer,^8^ it acts as tumour suppressor gene, while in gastric cancer it promotes migration and invasion of gastric cancer cells,^9^ suggesting that EGR1 may play the role of an oncogene in gastric cancer. EGR1 may act as both an activator and inhibitor of downstream target genes. The downstream molecules activated by EGR1 such as miR‐152,^10^ PTEN^11^ and Siva‐1,^12^ and the downstream molecules inhibited such as Stathmin,^13^ MEF2.^14^ EGR1 interacts with Snail,^15^ NF‐kappaB,^16^ Ref‐1^17^ to affect transcription. Bioinformatics analysis was used to analyse proteins bound to EGR1, and we identified DNA methyltransferase 3‐like protein (DNMT3L) as a candidate. DNMT3L plays a key role in gene silencing by functioning as a repressor by directly binding to the HDAC1 protein.^18^

## 2 MATERIALS AND METHODS

### 2.1 GC tissues and cell lines

Twenty‐two GC tumour tissue samples and the matched adjacent non‐malignant tissue samples were surgically obtained from the patients of the First Affiliated Hospital of Xi'an Jiaotong University. Informed consent was provided by all patients. This study was approved by the Medical Ethical Committee of the College of Medicine, Xi'an Jiaotong University. Human GC cell lines (SGC‐7901, MKN45 and BGC823) and the non‐malignant gastric epithelium cell line (GES‐1) were maintained in the Key Laboratory of Environment and Genes Related to Diseases at Xi'an Jiaotong University. All the cells were cultured in RPMI‐1640 medium with 10% foetal bovine serum in a humidified cell incubator with an atmosphere of 5% CO_2_ at 37°C.

### 2.2 RNA extraction and quantitative reverse transcription polymerase chain reaction

The TRIzol reagent (Invitrogen) was used according to manufacturer’s instructions to isolate RNA from the tissues and cells. The total RNA and PrimeScript RT reagent were used to generate cDNA according to manufacturer's instructions. quantitative reverse transcription polymerase chain reaction (qRT‐PCR) was performed using SYBR Green Master Mix (Takara) on a FTC‐3000TM System. β‐Actin and U6 were used as endogenous controls normalize mRNA and miRNA, respectively. Sequence information is listed here:

- miR‐195‐RT: 5′GTCGTATCCAGTGCGTGTCGTGGAGTCGGCAATTGCACTGGATACGACGCCAATA3′
- miR‐195‐F: 5′ATCCAGTGCGTGTCGTG3′
- miR‐195‐R: 5′TGCTTAGCAGCACAGAAA3′
- U6 RT: 5′CGCTTCACGAATTTGCGTGTCAT 3′
- U6‐F: 5′GCTTCGGCAGCACATATACTAAAAT 3′
- U6‐R: 5′CGCTTCACGAATTTGCGTGTCAT3′

### 2.3 Expression vector construction and transient transfection

The oligonucleotides of pre‐miR‐195 were synthesized and cloned into the EcoRI and HindIII sites of the pcDNA6.2‐GW vector (Invitrogen). The sequences of 3′‐ UTR of AKT3 were synthesized and cloned between the SacI and XhoI sites of the pmirGLODual‐Luciferase miRNA Target Expression Vector (Promega). Small interfering RNA (siRNA) against EGR1 and siRNA against AKT3 were purchased from GenePharma (GenePharma). SGC‐7901 and BGC‐823 cells were cultured in RPMI‐1640 medium with 10% foetal bovine serum for 24 hours. Then, jetPRIME^®^ from Polyplus‐transfection according to the manufacturer's instructions was used to transfect the pre‐miR‐195 overexpression vector, miR‐ctrl, miR‐195 inhibitor, inhibitor‐ctrl, EGR1‐ctrl, EGR1, si‐EGR1, si‐ctrl, si‐AKT3 or si‐ctrl into the cells. Sequence information is listed here (Shanghai GenePharma Co., Ltd):

- si‐AKT3‐S: 5′GGCAAGUGGAAAAUACUAUTT 3′
- si‐AKT3‐A: 5′ AUAGUAUUUUCCACUUGCCTT 3′
- si‐EGR1‐S: 5′ GTGACTGTTTGGCTTATAATT 3′
- si‐EGR1‐A: 5′ TTATAAGCCAAACAGTCACTT 3′
- siRNA‐ctrl‐S:5′UUCUCCGAACGUGUCACGUTT3′ and
- siRNA‐ctrl‐A, 5′ACGUGACACGUUCGGAGAATT 3′
- β‐actin‐F:5′CCAACCGCGAGAAGATGA3′
- β‐actin‐R: 5′CCAGAGGCGTACAGGGATAG 3′

### 2.4 MTT assay

SGC‐7901 and BGC‐823 cells were seeded in 96‐well plates. After 24 hours, they were transfected with the pre‐miR‐195 overexpression vector, miR‐195 inhibitor, EGR1 vector, si‐EGR1, si‐AKT3 or their respective controls. The cells were then incubated for 24, 48 and 72 hours. MTT solution (20 μL) was added to each well and cells were incubated for 4 hours at 37°C. After that, the supernatants were discarded, and formazan crystals were dissolved in 150 μL dimethylsulphoxide (DMSO) and the absorbance was measured.

### 2.5 Colony‐forming assays

SGC‐7901 and BGC‐823 cells were seeded in plates, then cells were transfected with either pre‐miR‐195 overexpression vector, miR‐ctrl, miR‐195 inhibitor, inhibitor‐ctrl, EGR1‐ctrl, EGR1 vector, si‐EGR1, si‐ctrl, si‐AKT3 or si‐ctrl. After 14‐day‐incubation, the cells were washed with phosphate‐buffered saline (PBS) and stained with crystal violet solution. Images of the colonies were obtained using Quantity One computer software (Bio‐Rad, Hercules).

### 2.6 Cell apoptosis assay

SGC‐7901 and BGC‐823 cells were seeded into 6‐well plates, and cells were collected 48 hours after transfection. Subsequently, cells were stained using the annexin‐V‐FITC/PI.

Apoptosis Detection Kit according to manufacturer’s instructions. Cell apoptosis was examined by flow cytometry (FACSort, Becton Dickinson). To estimate the nuclear morphology by DAPI staining, SGC‐7901 and BGC‐823 cells were seeded into 8‐well plates and transfected with pre‐miR‐195 overexpression vector, miR‐ctrl, miR‐195 inhibitor and inhibitor‐ctrl. Cells were washed with PBS, fixed with 4% paraformaldehyde for 15 minutes, and nuclei were stained with DAPI staining solution for 10 minutes in the dark. Images were acquired by a Nikon C2 Confocal Laser Microscope.

### 2.7 Chromatin immunoprecipitation assay

The binding of EGR1 to the miR‐195 promoter was detected by chromatin immunoprecipitation (ChIP). Protein/DNA complexes were obtained from BGC‐823 cells, which were cross‐linked with 1% formaldehyde for 15 minutes at room temperature, and quenched by adding glycine (0.125 mol/L) for 30 minutes. Cells were collected after rinsing two times with 5 mL of PBS and the nuclei were resuspended in Mg‐NI, Mg‐NIXP40, Ca‐NI (with 0.5 mol/L EGTA) and lysis buffer (with protease inhibitors). The samples were sonicated by a cell cracker and the chromatin was sheared into ~200 bp fragments. The sample was centrifuged to remove the insoluble material. 100 μl was used as input the lysates were divided into two parts and incubated with antibodies against EGR1 or IgG overnight at 4°C and then bound to protein G Sepharose (Invitrogen) for 2 hours at 4°C. The immunoprecipitates were consecutively washed twice byChIP lysis buffer, and finally TE buffer. The bound proteins were eluted from the beads by a solution containing 1% SDS and 0.1 mol/L sodium bicarbonate. Both the input and the samples were reverse cross‐linked with proteinase K for 8 hours at 65°C. DNA was isolated using phenol/chloroform (Invitrogen). Promoter binding was detected by PCR using primers spanning the upstream regions of the miR‐195 start sites. The primer sequences used are listed here:

- S: 5′ CCCAACCCAAGAAAAAAAACTGC3′
- A: 5′ CCGGGGGGAATCACTCCTCAAAG3′

### 2.8 Western blot analysis

Protein was extracted with the radioimmunoprecipitation assay cell lysis buffer (Wolsen). Equal amounts of proteins in each sample were separated by 10% SDS polyacrylamide gels and electrophoretically transferred to an activated polyvinylidene difluoride membrane. Subsequently, the membranes were blocked in 5% dry milk in Tris‐buffered saline containing 0.1% Tween. Then followed the incubation with the following primary antibodies at 4°C overnight: AKT3 (ProteinTech Group; diluted 1:1000), Bcl‐2 (Bioworld; diluted 1/500), Bax (Bioworld; diluted 1/500) and β‐actin (Santa Cruz Biotechnology; dilution 1:1000). After washing three times, the membrane was incubated with secondary antibodies for 1 hour at room temperature.

### 2.9 Co‐immunoprecipitation

Cellular proteins and protein complexes were extracted from SGC‐7901 and BGC‐823 cells by RIPA lysates. Then, the supernatant was added to the primary antibody. The antigen‐antibody complex was placed on a slowly rotating shaker at 4°C for overnight. The DynabeadsTM Protein G (Invitrogen) were washed two times with PBS, and a 50% protein G beads working solution was prepared. This 50% protein G beads solution was then added into the sample solution, and the sample was placed on a slowly rotating shaker at 4°C for 4 hours or overnight. The sample was then centrifuged, the supernatant was discarded, and the protein G beads were collected. The beads were thereupon washed, and the supernatant was collected for Western blot.

### 2.10 5‐aza‐dC treatment

The DNA methyltransferase inhibitor, 5‐aza‐dC (Sigma‐Aldrich; Merck KGaA, Darmstadt, Germany) was added at different concentrations (0.0, 2.0, 4.0, 6.0, 8.0 and 10.0 µmol/L) to SGC‐7901 and BGC‐823 cells for 48 hours 37°C. Then, TRIzol (Invitrogen; Thermo Fisher Scientific, Inc) was used according to manufacturer’s protocol to isolate RNA from SGC‐7901 and BGC‐823 cells. RT‐qPCR was performed to detect the miR‐195 expression.

### 2.11 Dual‐luciferase assay

HEK293 cells were seeded into 96‐well plates. After 24 hours, pmirGLO‐AKT3‐3′‐UTR vectors with wildtype or mutated miR‐195‐binding sites was cotransfected with pre‐miR‐195 into HEK293 cells. The pmirGLO vector was used as control. Firefly and Renilla luciferase activity was detected with the dual‐Luciferase Assay System (Promega) 24 hours post‐transfection according to manufacturer instructions.

- AKT3‐WT‐S5′CAAGGTCTCATGCTGTTGCTGCTAC3′
- AKT3‐WT‐A 5′ TCGAGTAGCAGCAACAGCATGAGACCTTGAGCT3′
- AKT3‐MT‐S5′CAAGGTCTCATGCTGTTGCACGTAC3′
- AKT3‐MT‐A5′ TCGAGTACGTGCAACAGCATGAGACCTTGAGCT3′

### 2.12 Statistical analysis

All data are presented as mean ± SEM. The Student's *t* test was used to evaluate differences between two groups. Data were considered to be statistically significant when *P* < .05.

## 3 RESULTS

### 3.1 The miR‐195 could inhibit proliferation and induce apoptosis in GC cells

**Figure 1:**
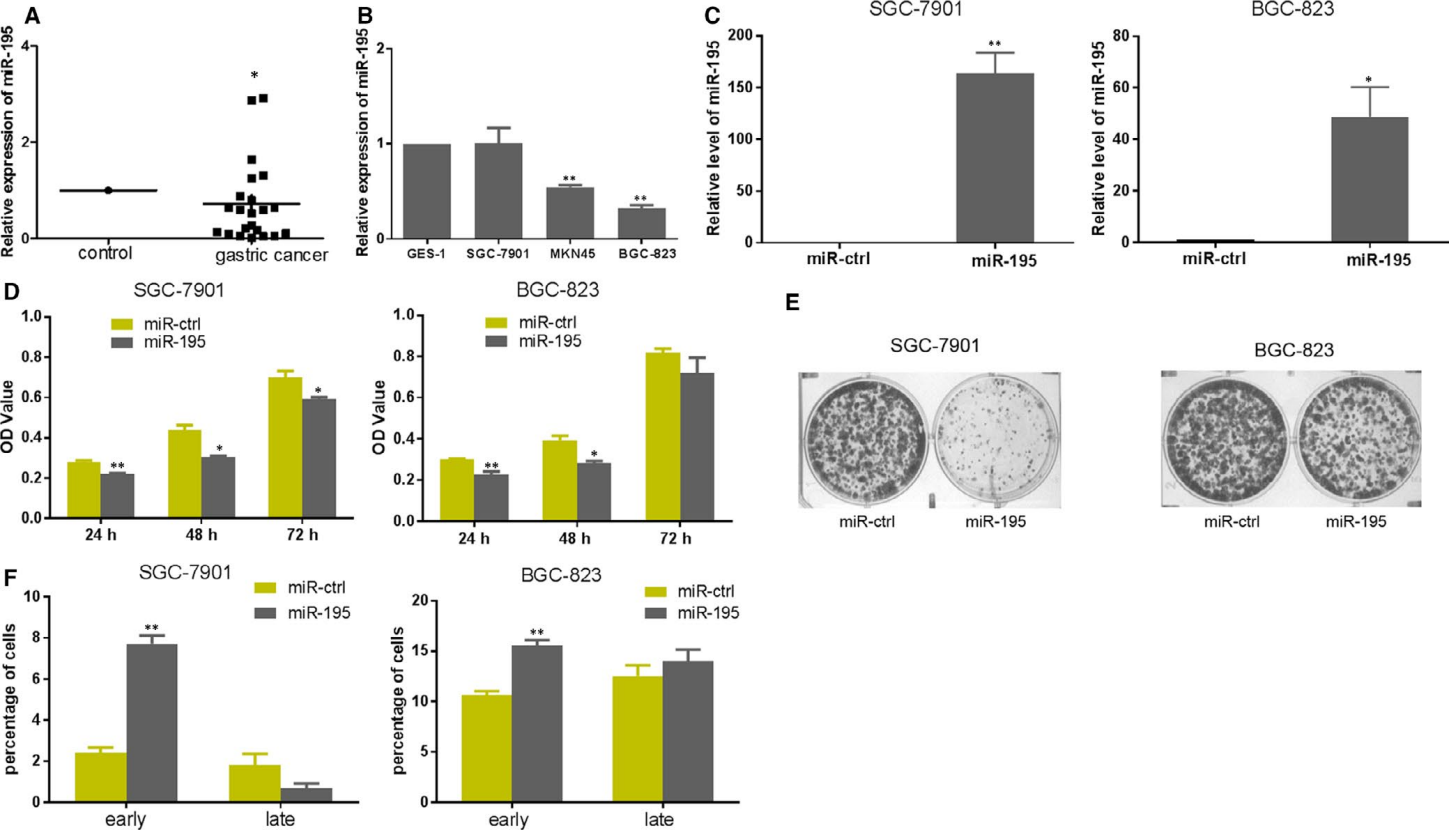
miR‐195 inhibits GC cells proliferation and induces apoptosis. A, qRT‐PCR was performed to analyse the expression of miR‐195 in 22 paired human gastric cancer and adjacent normal tissues. B, qRT‐PCR was performed to analyse the expression of miR‐195 in gastric cancer cells and normal gastric mucosal cells. C, qRT‐PCR was performed to analyse the expression of miR‐195 after SGC‐7901/BGC‐823 cells transfection with pre‐miR‐195 or miR‐ctrl. D, MTT assay of SGC‐7901/BGC‐823 cells transfected with miR‐195 or miR‐ctrl. E, Colony formation assays of SGC‐7901/BGC‐823 cells transfected with miR‐195 or miR‐ctrl. F, Apoptosis assay in SGC‐7901/BGC‐823 cells by annexin‐V/propidium iodide through flow cytometry after transfection with miR‐195 or miR‐ctrl (^*^ *P* < .05)

**Figure 2:**
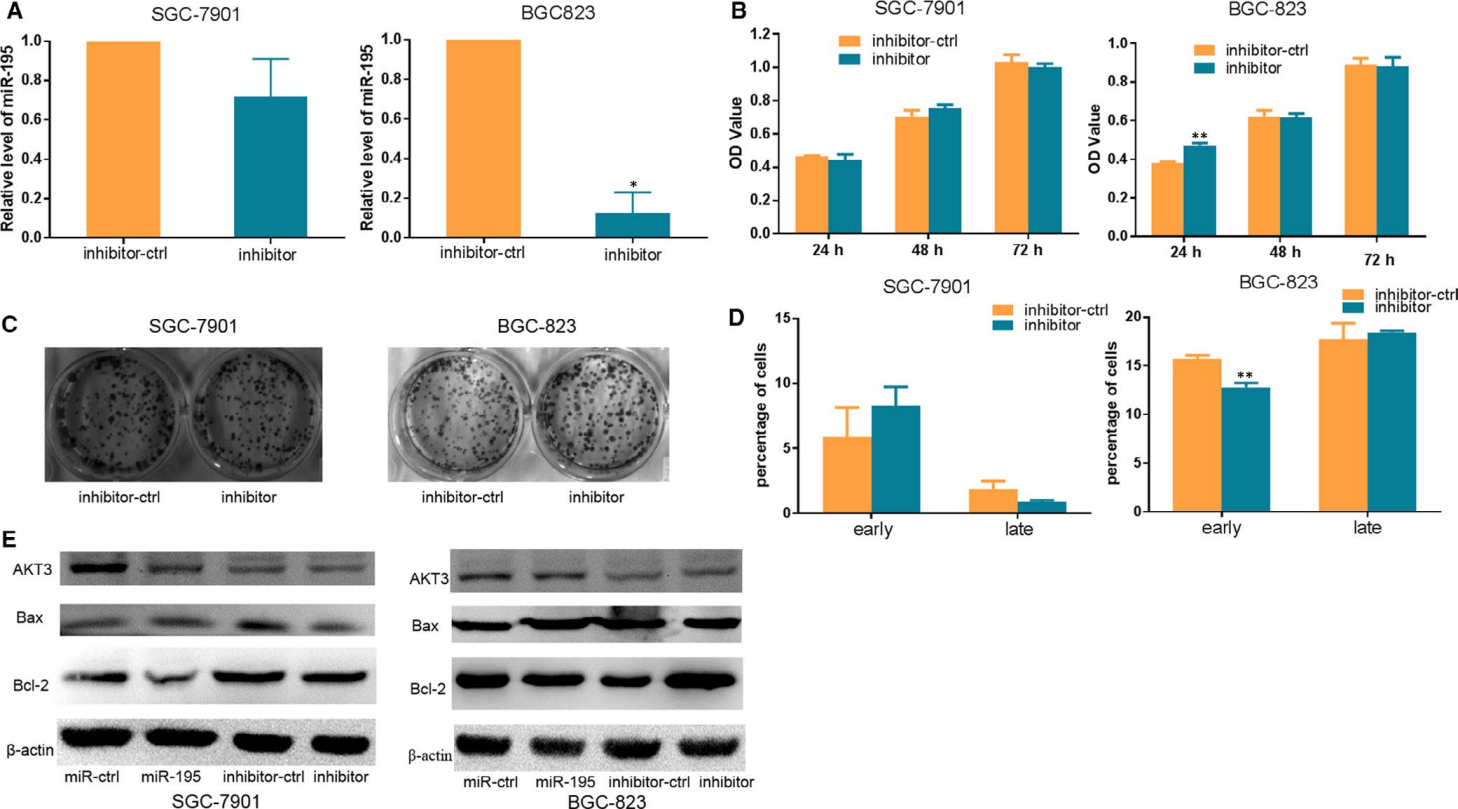
miR‐195 inhibitor promotes GC cells proliferation and inhibits apoptosis. A, qRT‐PCR was performed to analyse the expression of miR‐195 after SGC‐7901/BGC‐823 cells transfection with miR‐195 inhibitor or inhibitor‐ctrl. B, MTT assay of SGC‐7901/BGC‐823 cells transfected with miR‐195‐inhibitor or inhibitor‐ctrl. C, Colony formation assays of SGC‐7901/BGC‐823 cells transfected with miR‐195‐inhibitor or inhibitor‐ctrl. D, Apoptosis detection after miR‐195‐inhibitor or inhibitor‐ctrl transfection. E, Western blot of AKT3, Bcl‐2 and Bax after pre‐miR‐195, miR‐ctrl, miR‐195 inhibitor or inhibitor‐ctrl transfection in SGC‐7901/BGC‐823 cells (^*^ *P* < .05, ^**^ *P* < .01)

To explore the function of miR‐195 in gastric cancer, qRT‐PCR was performed to detect the expression of miR‐195 in GC and normal tissues. The results showed that miR‐195 was downregulated in GC tissues compared to normal tissues (Figure 1A). In addition, comparing the expression of miR‐195 in the GC cell lines (SGC‐7901, BGC‐823 and MKN45) with the GES‐1 cell line by qRT‐PCR, the results showed that miR‐195 was downregulated in MKN45 and BGC‐823 cells (Figure 1B). The qRT‐PCR was performed to detect the expression of miR‐195 after pre‐miR‐195 was transfected into SGC‐7901 and BGC‐823 cells, and the results revealed that the expression of miR‐195 was increased in cells transfected with pre‐miR‐195 compared with cells transfected with miR‐control (Figure 1C). The MTT assays and colony formation assays were used to investigate the effect of miR‐195 on cell proliferation, and the result revealed that overexpression of miR‐195 caused proliferation inhibition on cell growth and colony formation after transfection in SGC‐7901 and BGC‐823 cells (Figure 1D‐E). The proportion of apoptotic cells increased in cells transfected with pre‐miR‐195 compared with cells transfected with miR‐control (Figure 1F). It was observed that overexpression of miR‐195 caused apoptosis in SGC‐7901 and BGC‐823 cells (Figure S1).Western blot results for detection of protein expression of AKT3, Bcl‐2 and Bax verified that after pre‐miR‐195 and control vector transfection, the protein expression of AKT3 decreased in SGC‐7901 cells (Figure 2E). These data demonstrated that miR‐195 inhibited proliferation and induced apoptosis in GC cells, which indicated that miR‐195 acted as a tumour suppressor in GC.

### 3.2 Silencing the expression of miR‐195 could promote proliferation and repress apoptosis in GC cells

qRT‐PCR was performed to detect the transfection efficiency of miR‐195 inhibitor in SGC‐7901 and BGC‐823 cells, and the results showed that the expression of miR‐195 was decreased in cells transfected with miR‐195‐inhibitor compared with cells transfected with inhibitor‐control (Figure 2A). MTT assays were used to investigate the effect of miR‐195 inhibitor on cell proliferation, and the result revealed that miR‐195 inhibitor improved proliferation of BGC‐823 cells compared with cells transfected with inhibitor‐control (Figure 2B). Colony forming assays showed that miR‐195‐inhibitor‐transfected cells exhibited no obvious difference compared with inhibitor‐ctrl‐transfected cells (Figure 2C).The apoptosis assay showed that the miR‐195 inhibitor suppressed the early apoptosis of BGC‐823 cells compared with cells transfected with inhibitor‐control (Figure 2D). Western blot results for detection of protein expression of AKT3, Bcl‐2 and Bax verified that after miR‐195 inhibitor and inhibitor‐control transfection, the miR‐195 inhibitor decreased the protein expression of Bax (Figure 2E). These data demonstrated that silencing the expression of miR‐195 could induce proliferation and repress apoptosis in BGC‐823 cells.

### 3.3 EGR1 affects miR‐195 promoter activity in GC cells

**Figure 3:**
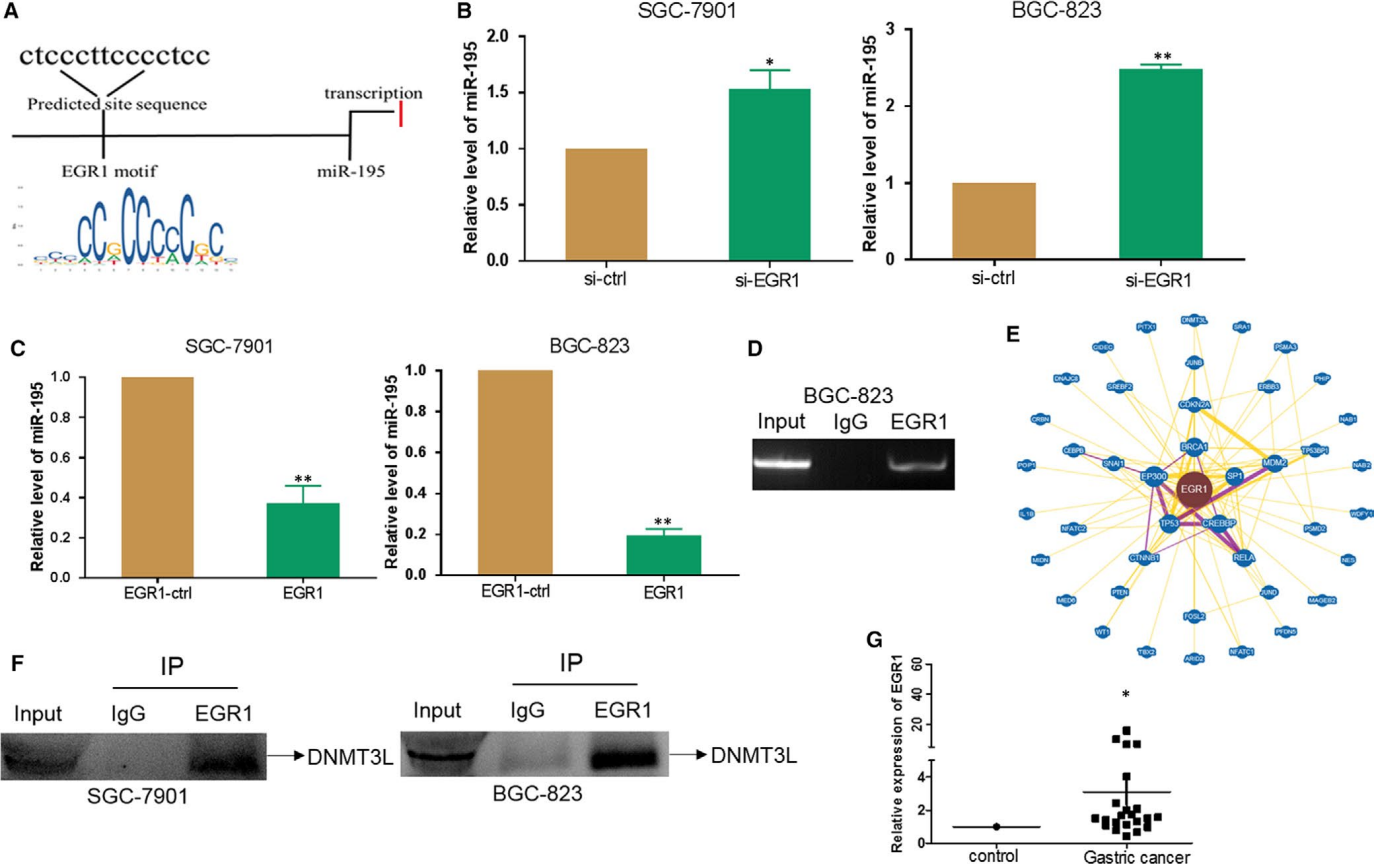
EGR1 represses miR‐195 promoter activity in gastric cancer cells. A, Schematic diagram of the miR‐195 promoter with one potential EGR1 response element B, qRT‐PCR was performed to examine the expression of miR‐195 after transfection with si‐EGR1 or si‐ctrl in SGC‐7901/BGC‐823 cells. C, The expression of miR‐195 after transfection with EGR1 or control vector in SGC‐7901/BGC‐823 cells. D, ChIP assays of EGR1 occupancy at the miR‐195 promoter. E, BioGrid software forecast of proteins that may form a complex with EGR1. F, Co‐IP assay of EGR1 and DNMT3L. G, The expression of EGR1 in paired human gastric cancer and adjacent normal tissues (^*^ *P* < .05, ^**^ *P* < .01)

**Figure 4:**
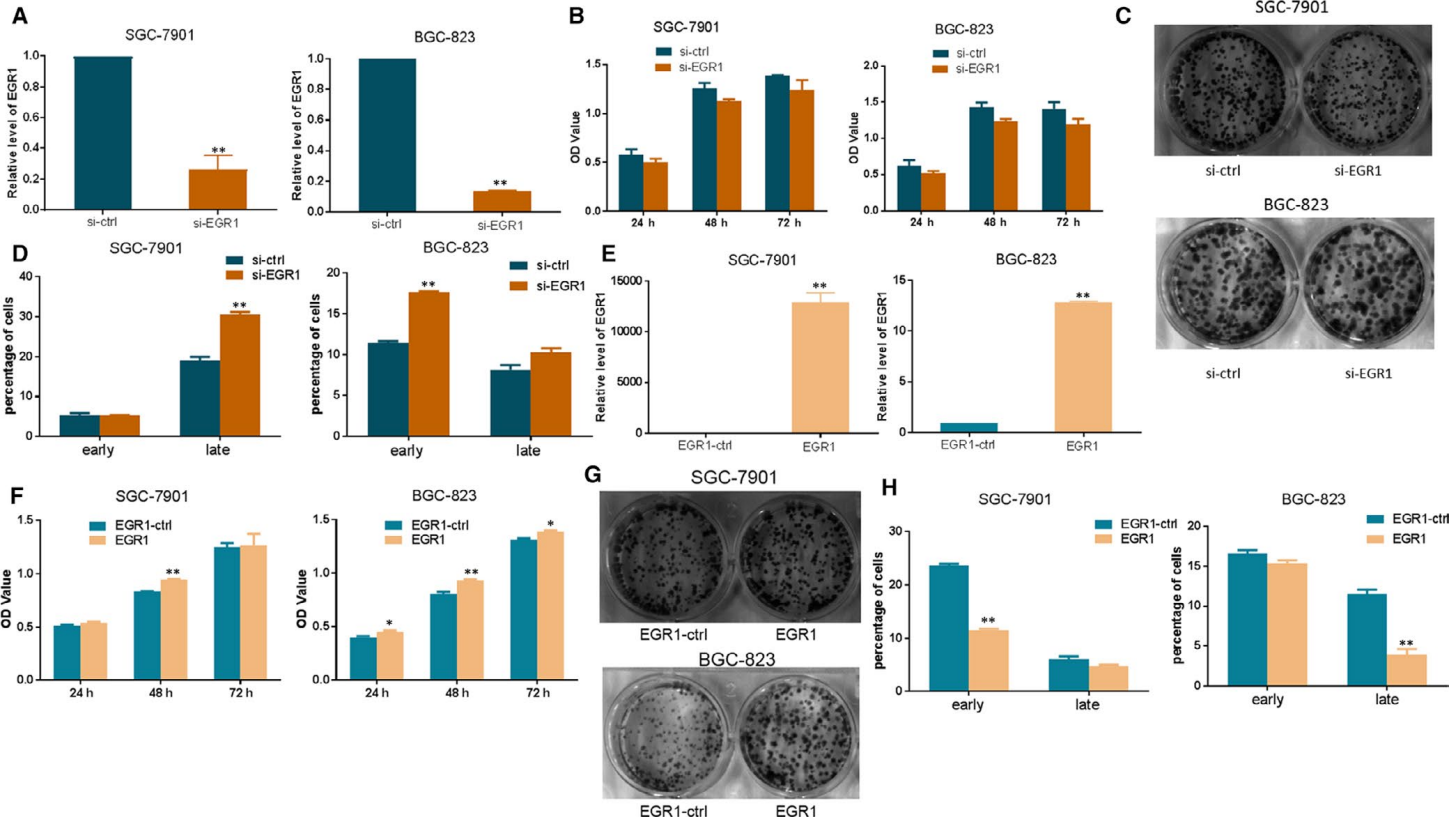
EGR1 affects apoptosis in GC cells. A, qRT‐PCR analysis of EGR1 expression after SGC‐7901/BGC‐823 cells transfected with si‐EGR1 or si‐ctrl. B, MTT assay of GC cells treated with si‐EGR1 or si‐ctrl. C, Colony formation assays of SGC‐7901/BGC‐823 cells transfected with si‐EGR1 or si‐ctrl. D, Apoptosis assay in SGC‐7901/BGC‐823 cells after transfection with si‐EGR1 or si‐ctrl. E, qRT‐PCR analysis of EGR1 expression after EGR1 or control vector transfection into SGC‐7901/BGC‐823 cells. F, MTT assay of GC cells treated with overexpression of EGR1. G, Colony formation assays of SGC‐7901/BGC‐823 cells transfected with EGR1 or EGR1‐ctrl. H, Apoptosis assays in SGC‐7901/BGC‐823 cells after transfection with EGR1 or EGR1‐ctrl (^*^ *P* < .05, ^**^ *P* < .01)

To explore the mechanism of downregulation of miR‐195 in GC, bioinformatic software was used to identify putative binding sequences at the miR‐195 promoter region. We found that EGR1 binding sites were located upstream of the miR‐195 gene (Figure 3A). We transfected si‐EGR1 into SGC‐7901 and BGC‐823 cells and subsequently performed qRT‐PCR to detect the expression of miR‐195. The result showed that si‐EGR1 silenced the expression of EGR1 (Figure 4A) and increased the expression of miR‐195 in SGC‐7901 and BGC‐823 cells (Figure 3B). Furthermore, the EGR1 overexpression vector was transfected into SGC‐7901 and BGC‐823 cells, and qRT‐PCR was performed to detect the corresponding expression of miR‐195. The result showed that EGR1 vector indeed generated higher expression of EGR1 (Figure 4E) and suppressed the expression of miR‐195 (Figure 3C). ChIP analysis revealed that the EGR1 protein bound to the putative binding site upstream of miR‐195 in BGC‐823 cells (Figure 3D). The mechanism by which EGR1 exerted transcriptional repression, possibly by forming a complex, is still unclear. The BioGrid software forecast detected that EGR1 may interact with DNMT3L (Figure 3E). In order to detect whether EGR1 and DNMT3L form a complex, we performed the co‐IP assay. The results showed that EGR1 and DNMT3L indeed could form a complex (Figure 3F). Because DNMT3L acts in DNA methylation, we used 5‐aza‐dC to treat SGC‐7901 and BGC‐823 cells and found that 5‐aza‐dC increased the expression of miR‐195 in BGC‐823 cells (Figure S2). The qRT‐PCR was performed to detect the expression of EGR1, and the results showed that the expression of EGR1 mRNA levels was higher in GC tissues compared with their respective non‐tumour tissue (Figure 3G). These findings revealed that EGR1 and DNMT3L formed a complex that may act to repress transcription.

### 3.4 EGR1 affects the apoptosis of gastric cancer cells

The qRT‐PCR results demonstrated that si‐EGR1 significantly silenced the expression of EGR1 (Figure 4A), while the MTT assay showed that silencing the expression of EGR1 exhibited suppressing of the proliferation trend in SGC‐7901 and BGC‐823 cells. However, there were no significant differences (Figure 4B). Colony‐forming assays showed that si‐EGR1‐transfected cells exhibited no obvious difference compared with si‐ctrl‐transfected cells (Figure 4C). Meanwhile, silencing the expression of EGR1 induced apoptosis (Figure 4D). Upon transfection of the EGR1 vector into SGC‐7901 and BGC‐823 cells, the qRT‐PCR results showed that the vector indeed generated higher expression of EGR1 (Figure 4E). The overexpression of EGR1 promoted the proliferation of SGC‐7901 and BGC‐823 cells (Figure 4F). Cells transfected with EGR1 exhibited more colonies compared with EGR1‐ctrl in BGC‐823 cells (Figure 4G). The apoptosis assay demonstrated that overexpression of EGR1 suppressed the early apoptosis in SGC‐7901 cells, compared with cells transfected with the control vector (Figure 4H).

### 3.5 AKT3 is a direct target of miR‐195 and silencing the expression of AKT3 could inhibit proliferation and induce apoptosis in GC cells

**Figure 5:**
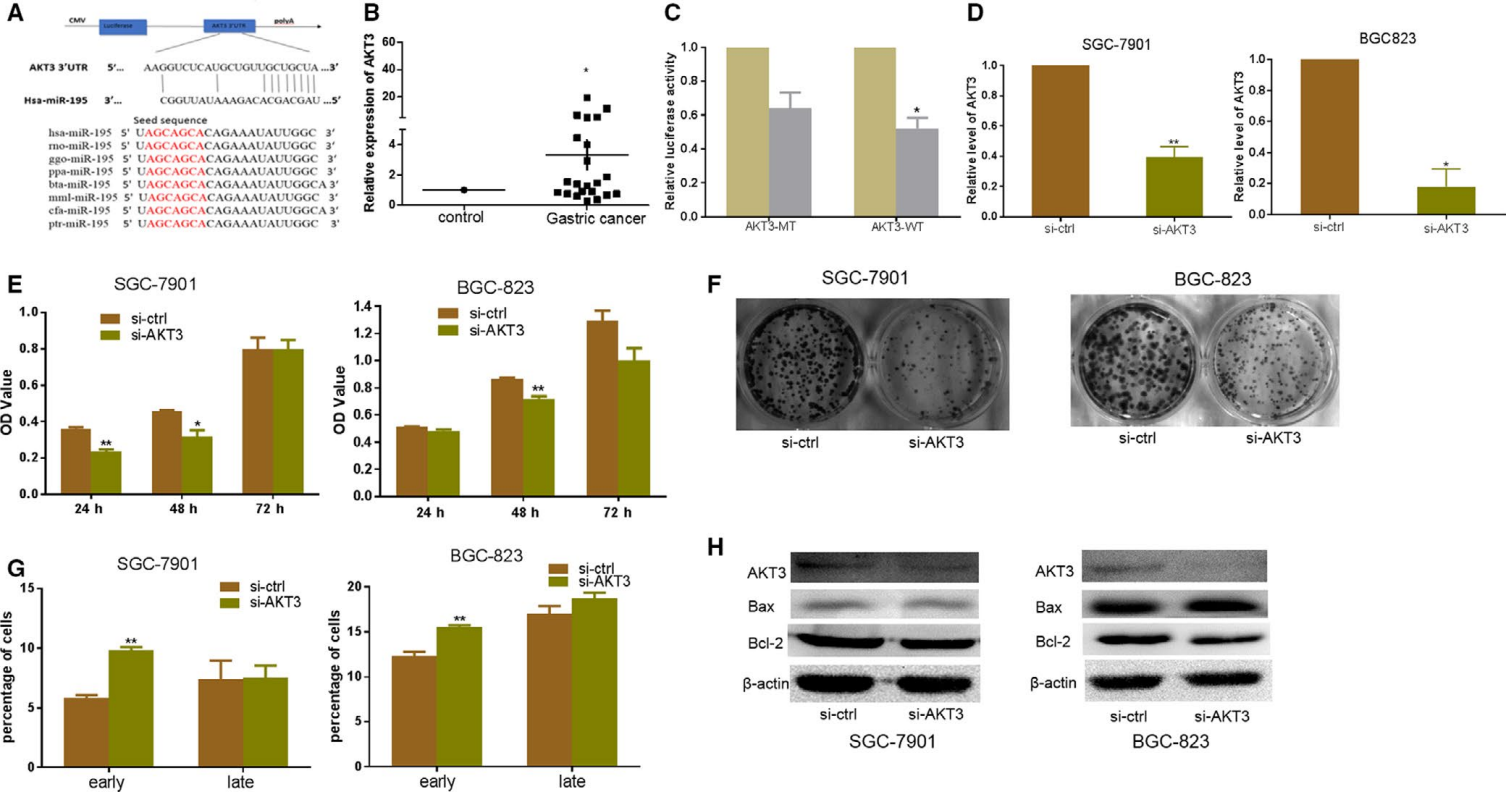
AKT3 is a direct target of miR‐195 and silencing the expression of AKT3 inhibit GC cells proliferation and induce apoptosis. A, The miR‐195 has the binding sites of the 3′‐UTR of AKT3 and the sequence of miR‐195 among different species. B, The expression of AKT3 in GC tissues compared with the respective non‐malignant tissue. C, Luciferase assay of the pre‐miR‐195 cotransfected with pGLO‐AKT3 wild‐type or pGLO‐AKT3 mutant vector into HEK293 cells, and GLO transfected with pre‐miR‐195 in HEK293 cells as control. D, The expression of AKT3 was determined after transfection with si‐AKT3 or si‐ctrl in SGC‐7901/BGC‐823 cells (E) MTT assay of GC cells treated with si‐AKT3 or si‐ctrl. F, Colony formation assays of SGC‐7901/BGC‐823 cells transfected with si‐AKT3 or si‐ctrl. G, Apoptosis assay of silenced AKT3 in SGC‐7901/BGC‐823 cells. H, Western blot of AKT3, Bcl‐2 and Bax after silencing AKT3 in SGC‐7901/BGC‐823 cells (^*^ *P* < .05, ^**^ *P* < .01)

The miRNA target prediction program was used to search for miR‐195 target genes. We found a well‐matched miR‐195 binding site at the AKT3 3′‐UTR. The sequence of miR‐195 was highly conserved among species (Figure 5A). The expression of AKT3 mRNA levels was higher in GC tissues compared with their respective non‐tumour tissue (Figure 5B). To determine whether AKT3 was a direct target of miR‐195, the wt/mut AKT3 3′‐UTR was transfected along with pre‐miR‐195 into HEK293 cells, and GLO was transfected with pre‐miR‐195 in HEK293 cells for control. After 24 hours, luciferase activity was markedly reduced in cells transfected with wt‐AKT3 3′‐UTR in comparison with control. The luciferase activity has no significant change in cells transfected with mt‐AKT3 3′‐UTR compared with cells treated with control (Figure 5C). To examine the role of AKT3 in GC progression, we silenced the expression of AKT3 by small interfering RNA (siRNA) against AKT3. The qRT‐PCR results showed that silencing AKT3 significantly downregulated the expression of AKT3 expression in both SGC‐7901 and BGC‐823 cells (Figure 5D). MTT assays and colony formation assays showed that silencing of AKT3 resulted in significant proliferation inhibition on cell growth and colony formation in BGC‐823 and SGC‐7901 cells (Figure 5E‐F). Flow cytometry was used to detect the effect of silencing AKT3 on cell apoptotic activity, and found that silencing AKT3 significantly increased the proportion of early apoptotic cells compared with GC cells transfected with the si‐control (Figure 5G). To verify the effect of silencing AKT3 on the protein level, we adopted Western blot analysis to detect the protein expression of AKT3, Bcl‐2 and Bax. The results verified that after si‐AKT3 transfection, the proteins AKT3 and BCL‐2 were decreased compared with transfected si‐control, while the protein expression of Bax was significantly upregulated in BGC‐823 cells (Figure 5H).

### 3.6 EGR1 mediates miR‐195 affect the GC progression by targeting AKT3

**Figure 6:**
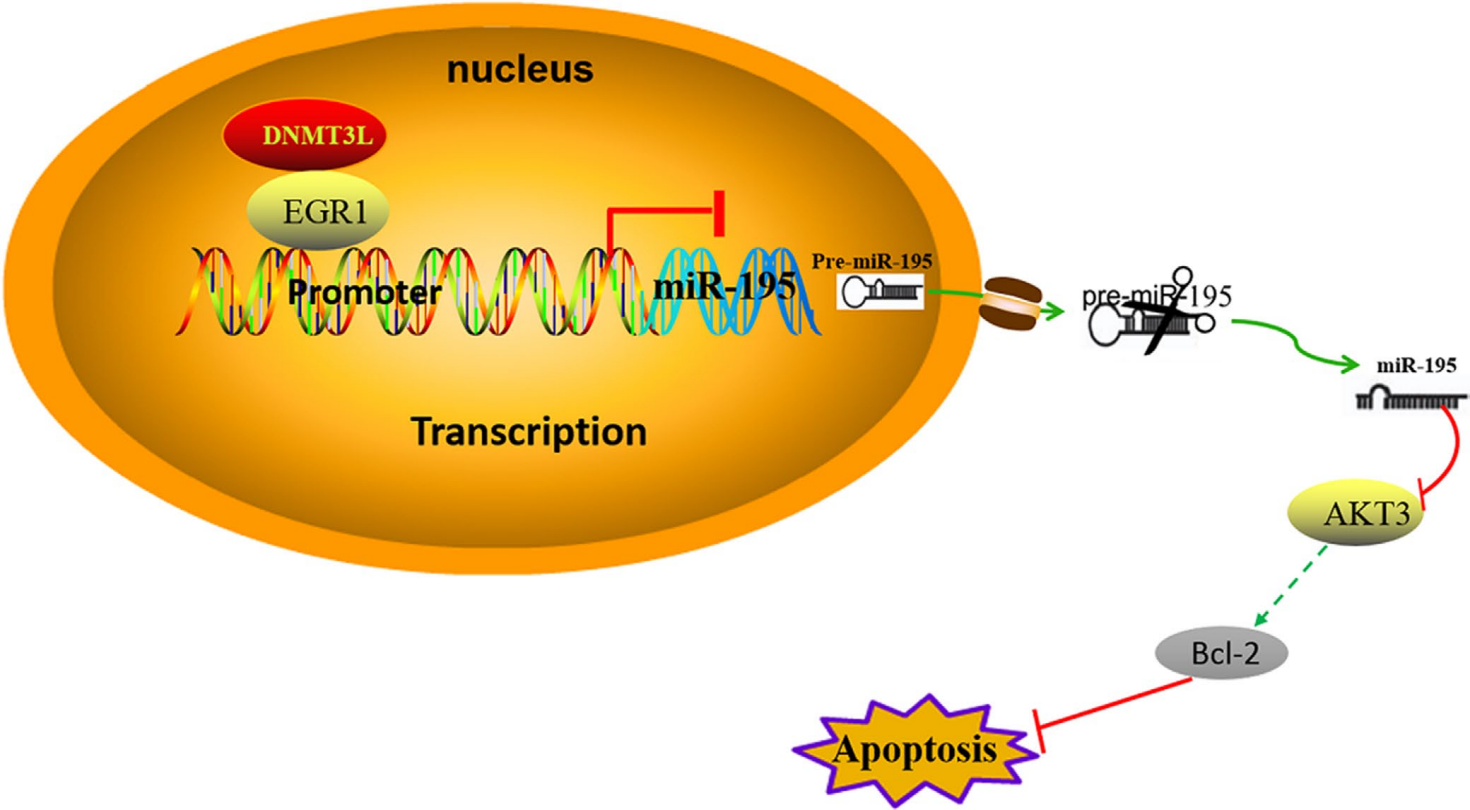
miR‐195 mediated by EGR1/DNMT3L functions as a tumour suppressor by targeting AKT3 in the development of gastric cancer. EGR1 binds to the miR‐195 promoter and EGR1/DNMT3L form a complex, inhibiting the expression of miR‐195. miR‐195 affects apoptosis of GC cells by targeting AKT3

EGR1 bound to the promoter sequence of miR‐195, repressing its expression. Moreover, EGR1 formed a complex with DNMT3L, which might be the reason for EGR1 acting as a transcriptional inhibitor. Repressed expression of miR‐195 promoted the GC cell progression, and inhibits the cell apoptosis by targeting AKT3 (Figure 6).

## 4 DISCUSSION

MicroRNAs are a class of highly conserved endogenous non‐coding RNA with 16‐22 nucleotides, which mainly through complementary action with the 3′‐UTR of target gene mRNA inhibit or decrease protein expression. The miRNAs are abnormally expressed in many malignant tumours in humans. miR‐195 is a member of the miRNA family, and it is located on chromosome 17 and involved in colorectal cancer,^19^ cervical cancer,^20^ prostate cancer,^21^ gastric cancer,^22^ liver cancer^23^ and many others. Bioinformatics Software UCSC Genome Browser Home and The JASPAR database analysis found that EGR1 is located upstream of the miR‐195. In this study, bioinformatic analyses suggested that EGR1 targets the upstream region of miR‐195. Moreover, our study showed that EGR1 was upregulated in GC tissues compared to their control. Furthermore, overexpression of EGR1 increased the proliferation of GC cells and suppressed their apoptosis. This implied that EGR1 may function as an oncogene in GC development. Our results of the ChIP assay demonstrated that EGR1 bound to the miR‐195 promoter and that overexpression of EGR1 inhibited the expression of miR‐195. These results indicated that EGR1 inhibited the transcription of miR‐195. However, the mechanism by which EGR1 acts as a transcriptional repressor is still unclear. BioGrid software was used to forecast which protein could form a complex with EGR1. DNMT3L was identified as one of the candidates. Thus, we conducted co‐IP assays to prove that EGR1 and DNMT3L indeed do form a complex and that DNMT3L acted primarily by regulation of the DNA methyltransferase.

The DNMT family has five members: DNMT1, DNMT2, DNMT3A, DNMT3B and DNMT3L. DNMT3L is one of the DNA methyltransferases (DNMTs) that acts primarily through regulation of the DNA methyltransferase. DNA methylation is the main method of genome modification in eukaryotic cells during growth and development. Abnormal DNA methylation could cause tumours due to gene transcription abnormalities. As one of the epigenetic mechanisms, methylation of the CPG island occurs in the promoter region of the tumour suppressor gene, resulting in gene silencing.

DNA methyltransferase could promote tumour development through aberrant methylation of tumour suppressor genes. For example, in gastrointestinal stromal tumours, DNMT1, DNMT2, DNMT3B and DNMT3L are highly expressed compared to non‐tumour tissues,^24^ and DNMT3L in testicular tumours is specifically expressed.^25^ DNMT3L is a key factor affecting cell differentiation and tumour formation, and early studies have confirmed its transcriptional repression.^18^ A previous study found that transcription factors such as FOS, MAFK, E2F3 and EGR1 could be interacted with DNMT3L through transcription factor array experiments, further suggesting that EGR1 may form a complex with DNMT3L.^26^

Serine/threonine‐specific protein kinase 3, AKT3, is a member of AKT family. There are three different AKT isoforms, AKT1, AKT2 and AKT3.^27^ AKT is an important signalling pathway in regulating several cellular functions including nutrient metabolism, cell growth, apoptosis and survival.^28^ AKT3 has been reported to be involved in cancer progression and plays important role in the regulations of various human cancers associated with miRNAs to modulate human cancers.^29^, ^30^, ^31^

Combining experimental results with bioinformatics analysis, we speculate that miR‐195 targets AKT3 to induce apoptosis in gastric cancer cells.

In conclusion, EGR1and DNMT3L formed a complex, which may be the reason why EGR1 was capable of inhibiting transcription of miR‐195. Moreover, overexpression of miR‐195 promoted apoptosis in gastric cancer cells by targeting AKT3, suggesting miR‐195 acted as a tumour suppressor gene in the development of gastric cancer.

## CONFLICT OF INTEREST

The authors declare there is no conflict of interest.

## AUTHOR'S CONTRIBUTION

YY and CH performed the experiments and wrote the manuscript. FW, JZ, RFS, FL, YLL, SEC, LMW, XFW and LYL designed the experiments and analysed the data.

## Data Availability

The data used to support the findings of this study are available from the corresponding author upon request.
