# Supplementary figures and images for: EGR1 interacts with DNMT3L to inhibit the transcription of miR‐195 and plays an anti‐apoptotic role in the development of gastric cancer
Source: J Cell Mol Med. 2019 Sep 12;23(11):7372–81. doi: 10.1111/jcmm.14597 (PMC6815817; doi:10.1111/jcmm.14597)

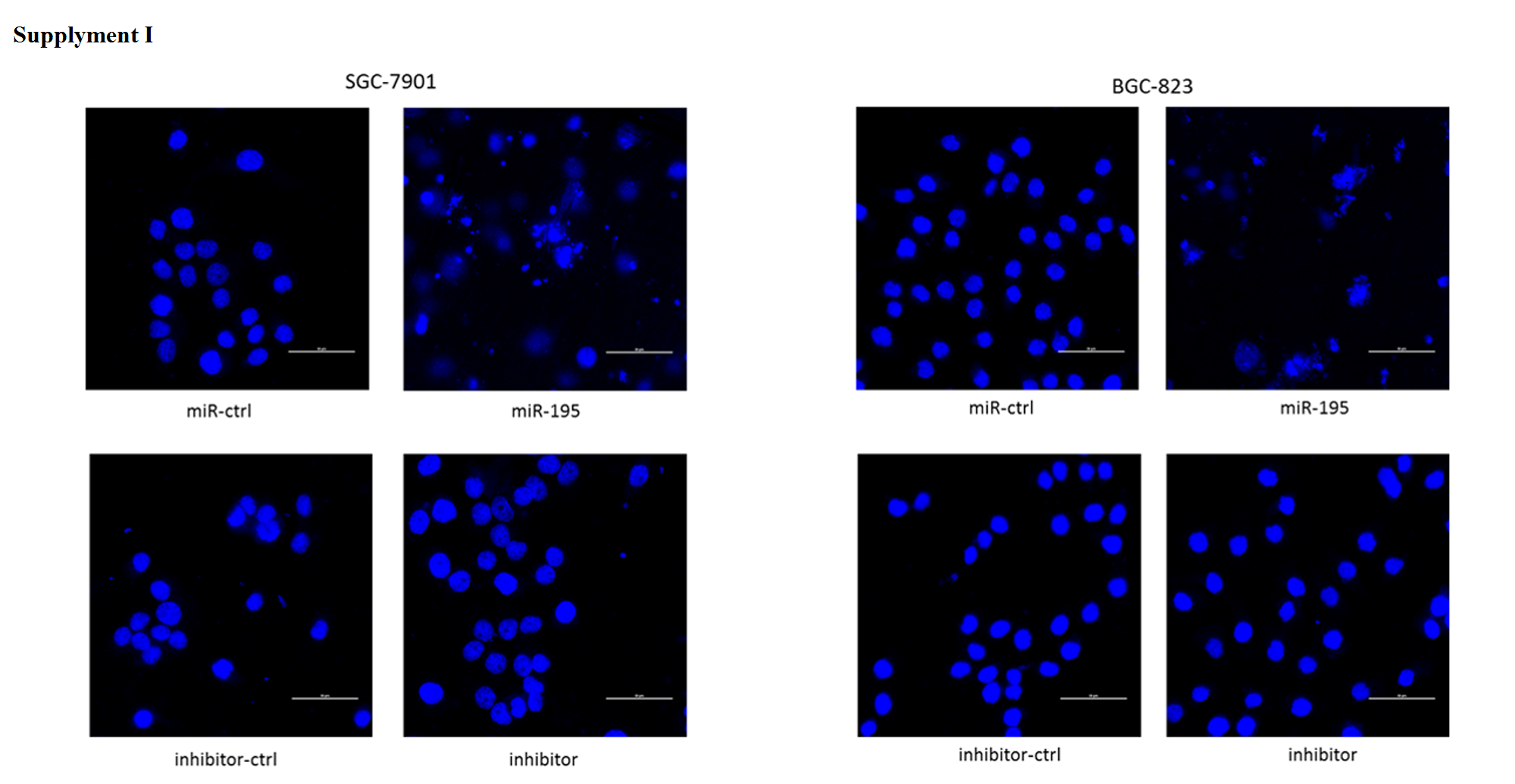

Supplement: Supplementary file 1 [file JCMM-23-7372-s001.tif]

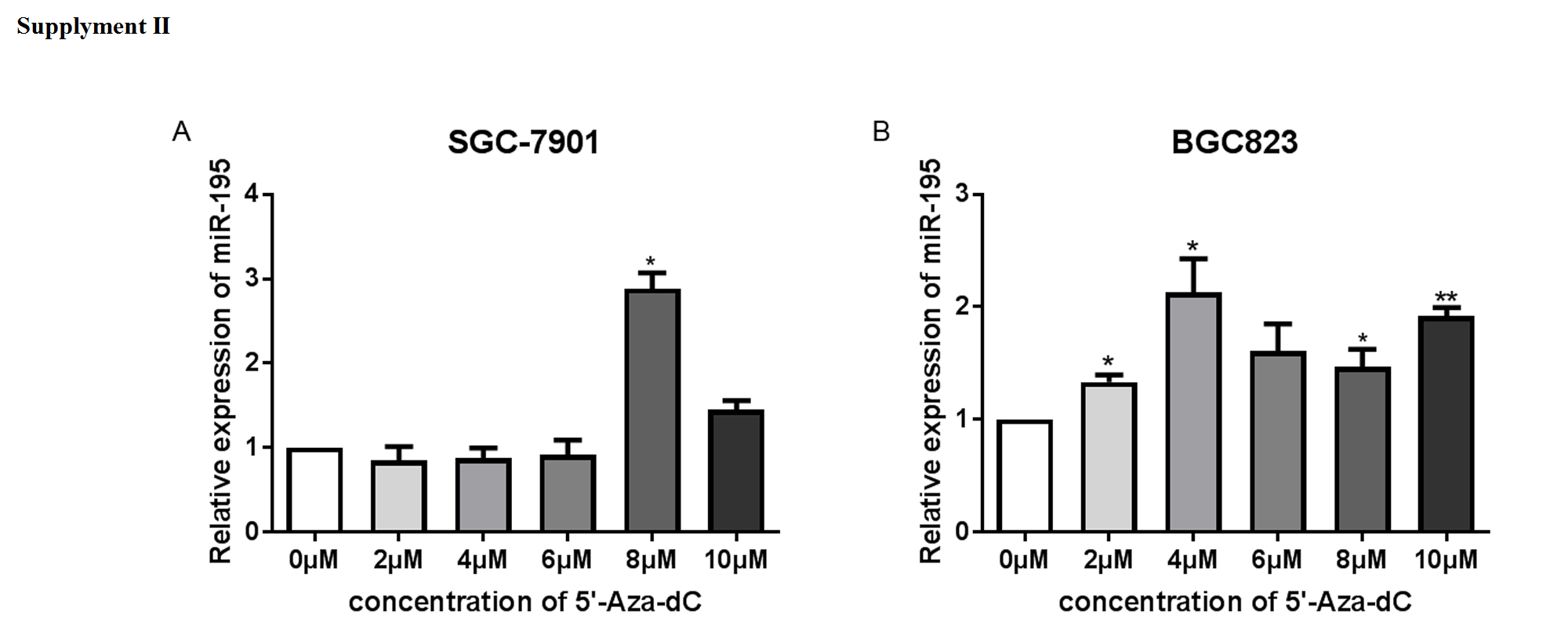

Supplement: Supplementary file 2 [file JCMM-23-7372-s002.tif]
